# Innate Lymphoid Cells: Emerging Players in Pancreatic Disease

**DOI:** 10.3390/ijms23073748

**Published:** 2022-03-29

**Authors:** Saimeng Shi, Longyun Ye, Kaizhou Jin, Zhiwen Xiao, Xianjun Yu, Weiding Wu

**Affiliations:** 1 Department of Pancreatic Surgery, Fudan University Shanghai Cancer Center, Shanghai 200032, China; shisaimeng@fudanpci.org (S.S.); yelongyun@fudanpci.org (L.Y.); jinkaizhou@fudanpci.org (K.J.); xiaozhiwen@fudanpci.org (Z.X.); 2 Department of Oncology, Shanghai Medical College, Fudan University, Shanghai 200032, China; 3 Shanghai Pancreatic Cancer Institute, Shanghai 200032, China; 4 Pancreatic Cancer Institute, Fudan University, Shanghai 200032, China

**Keywords:** innate lymphoid cells, diabetes mellitus, pancreatitis, pancreatic cancer

## Abstract

Common pancreatic diseases have caused significant economic and social burdens worldwide. The interstitial microenvironment is involved in and plays a crucial part in the occurrence and progression of pancreatic diseases. Innate lymphoid cells (ILCs), an innate population of immune cells which have only gradually entered our visual field in the last 10 years, play an important role in maintaining tissue homeostasis, regulating metabolism, and participating in regeneration and repair. Recent evidence indicates that ILCs in the pancreas, as well as in other tissues, are also key players in pancreatic disease and health. Herein, we examined the possible functions of different ILC subsets in common pancreatic diseases, including diabetes mellitus, pancreatitis and pancreatic cancer, and discussed the potential practical implications of the relevant findings for future further treatment of these pancreatic diseases.

## 1. Introduction

The pancreas is a glandular organ comprised of endocrine and exocrine parts, with a central role in energy homeostasis and metabolism. At a global level, diseases intrinsic to or associated with the pancreas have caused a major economic and social burden. Diabetes mellitus (DM, or diabetes) entered the top 10 global causes of death by 2019 [1], leading to over 4 million deaths a year [2]. Latest data from the International Diabetes Federation (IDF) show that there were 463 million people living with DM in 2019 and this figure is expected to be 700 million by 2045 [2,3]. Pancreatic cancer (PC) is one of the leading causes of cancer death worldwide, and its characteristics of rapid progression, early metastasis and late diagnosis make it the recognized “king of cancer”, with a five-year survival rate of only 9% [4,5].

The interstitial microenvironment is involved in and plays a crucial part in the occurrence and progression of pancreatic diseases. Regardless of whether they are benign or malignant pancreatic diseases, the interstitial microenvironment, in addition to the diseased cells themselves, is involved and plays a crucial role [6,7,8,9,10]. Immune cells are a vital player in the interstitial microenvironment, and the role of common subsets of lymphocytes, macrophages and dendritic cells (DCs) in pancreatic health and diseases has been studied.

Innate lymphoid cells (ILCs) are a heterogeneous population of lymphocytes, and their discovery has greatly expanded our understanding over the past 10 years [11,12]. ILCs are produced in the early stages of immune system formation and are mostly tissue-resident cells, exhibiting a tissue-specific subset distribution, phenotype, and functional regulation [13,14,15]. They are strategically located in the barrier tissues of the body, such as the skin, the mucosa of the digestive tract and respiratory tract [16,17,18,19]. When these sites are infected or damaged, ILCs sense them and react promptly to the environmental stress signals. They orchestrate acute inflammation to promote immunity to infection and facilitate the resolution of inflammation and tissue repair, as well as building bridges between the innate and adaptive immune systems, which processes are mainly mediated by their secreting various types of downstream signaling molecules. In addition to the typical immune processes, ILCs have also been demonstrated to be involved in processes not traditionally linked to the immune system, such as regulating metabolic homeostasis, epithelial differentiation and integrity [20,21,22,23].

In recent years, emerging evidence has identified the existence of ILCs in the pancreas, and all ILC subsets have been identified. ILCs resident in the pancreas, as well as in other tissues, may play an important role in the occurrence and progression of pancreatic diseases and will therefore be expected to be applied in the prevention and treatment of these diseases.

## 2. A Fundamental Overview of ILCs: Phenotype and Functions

Innate lymphoid cells (ILCs) represent a heterogeneous population of non-B/non-T lymphocytes and they are defined mainly by three unique features: (1) their lymphoid morphology; (2) their lack of genetically rearranged antigen receptors; and (3) their deficiency of cell-surface markers expressed in other immune cell types, such as myeloid cells and dendritic cells.

As a family of innate immune effector cells, ILCs have important roles in tissue homeostasis, metabolism, morphogenesis, repair and regeneration [20,21,22]. In addition to barrier tissues, such as the intestinal mucosa, lungs and skin, ILCs have also been found in adipose tissue (AT) and parenchymal organs. Such as the kidney and liver [24,25,26,27]. When these sites are infected or damaged, ILCs react promptly to environmental stress signals and produce an array of cytokines, promoting the resolution of inflammation and facilitating tissue repair [20]. In addition, ILCs have a direct complex impact on the adaptive immune response and build bridges between the innate and adaptive immune systems [23,28,29]. ILCs are also involved in processes not traditionally linked to the immune system, such as regulating metabolic homeostasis, epithelial differentiation and integrity [22]. However, uncontrolled activation and proliferation of ILCs can also lead to severe inflammation and damage [30,31].

**Figure 1:**
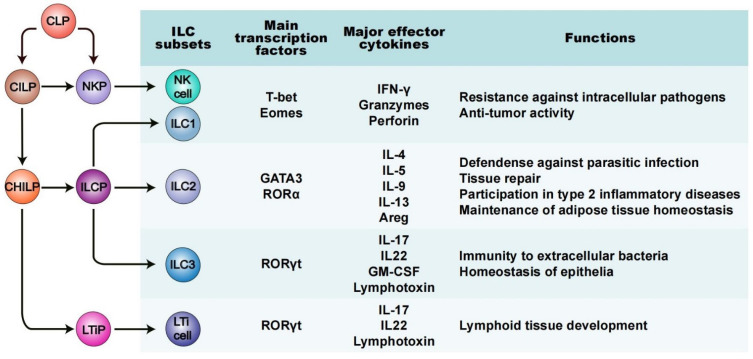
Development and the main features of ILC subsets. ILCs are derived from common lymphoid progenitors (CLPs) found within fetal liver and adult bone marrow. Dedicated TFs restrain B and T cell fates but guide the development of different ILC subsets. CLPs develop into common lymphoid progenitors (CILPs); then, through a series of transcriptional regulation processes, CILPs differentiate into natural killer cell precursor (NKPs) or common helper innate lymphoid progenitor (CHILPs), and the latter give rise to innate lymphoid cell precursor (ILCPs) and lymphoid tissue inducer progenitor (LTiPs). Each kind of precursor cell involves a branch in the ILC family. Based on the differential development trajectories and functions, the ILC family is categorized into five groups: NK cells, ILC1s, ILC2s, ILC3s and LTi cells. Each ILC subset secretes different effector cytokines that promote important physiological or pathological reactions.

Like all known lymphoid lineages, ILCs originate and develop from common lymphoid progenitors (CLPs) found within fetal liver and adult bone marrow (Figure 1) [32,33]. Based on their differential development trajectories and functions, the ILC family is categorized into five subsets: natural killer cells (NK cells, or “killer” ILCs), “helper” ILCs (ILC1s, ILC2s and ILC3s) and lymphoid tissue inducer (LTi) cells (Figure 1) [11,12,34]. Many current views regard ILCs as the innate counterpart of helper T cells, due to their strong similarities in the production and output of signature cytokines, and in the expression and production of several key transcription factors (TFs) [35,36]. ILC1s mirror CD4^+^ T helper (Th)1 cells, ILC2s mirror Th2 cells, ILC3s mirror Th17 cells, while NK cells represent the innate counterpart of CD8^+^ cytotoxic T cells [12,37]. Despite these similarities, ILCs’ unique epigenetic and transcriptional programs, as well as their crucial impact on health and disease, imply their nonredundant roles [38,39].

NK cells were first discovered in mice and humans in 1975 [40]. They are derived from natural killer cell precursor (NKP) cells, dependent on and characterized by the expression of the TFs Tbx21 (T-bet) and eomesodermin (Eomes) [41,42]. NK cells circulate in the bloodstream and are the counterpart of CD8^+^ cytotoxic T cells. They react to tumors and intracellular pathogens, producing interferon-γ (IFN-γ), granzymes and perforin, to kill tumor cells or normal cells infected by the virus [43,44]. ILC1s are the innate counterpart of Th1 cells and, resembling NK cells, are also involved in the immune response to tumor cells and intracellular pathogens, such as viruses and certain bacteria [45,46]. There are some common features between ILC1s and NK cells, such as requiring either T-bet, Eomes, or both, to achieve development and expressing IFN-γ as their principal cytokine output [47,48]. The difference is their discrepant developmental pathways and functional features. NK cells develop via NKPs, while ILC1s, like the other two “helper” ILCs, develop via ILCPs. In addition, ILC1s are tissue-resident cells and express only low levels of perforin, with less cytotoxicity [13,49]. ILC2s are defined by the expression of TFs GATA-binding protein 3 (GATA3) and retinoic acid receptor-related orphan receptor α (RORα), as well as the output of Th2 cytokines, including interleukin (IL)-4, IL-5, IL-9, IL-13 and the epidermal growth factor amphiregulin [50,51]. In addition to showing a response to parasitization [52], ILC2s are also involved in tissue repair and metabolic processes by type 2 immune responses [53,54,55]. ILC3s are the innate counterpart of Th17 cells. In mice, ILC3s rely on the TFs RORγt to develop and perform their functions and produce cytokines IL-17 and either IL-22, granulocyte macrophage colony-stimulating factor (GM-CSF, also known as Csf2), lymphotoxin, or a combination [12,56,57,58]. ILC3s are abundant in mucosal tissue, where they perform the innate immune response to extracellular pathogens and develop immune tolerance to intestinal symbionts. Thus, ILC dysfunction may lead to inflammatory diseases in mucosal-related tissues. LTi cells originate from CLPs via LTiPs and are strictly dependent on RORγt [59]. During the stage of embryonic development, LTi cells produce the cytokines of lymphotoxin, which play a key role in the formation of Peyer’s patches as well as secondary lymph nodes [60,61].

Recent evidence suggests the existence of ILCs in the pancreas, and all ILC subsets have been identified. As the role and functions of NK cells in the pancreas have been reviewed in detail elsewhere [62,63,64,65], there is only a brief discussion of their role in some parts. This paper mainly reviews the possible functions of “helper” ILC subsets in common pancreatic diseases, including DM, pancreatitis and PC, concentrating on the potential impact of ILCs on the occurrence and progression of these diseases and their contributions to the prevention, diagnosis and treatment.

## 3. ILCs in Diabetes Mellitus

Diabetes mellitus (DM, or diabetes) is recognized as one of the most significant and daunting public health challenges of the twenty-first century. The latest data from the IDF show that there were 463 million people living with DM in 2019 and this figure is expected to be 700 million by 2045 [2,3]. DM has devastating effects on individuals, societies and countries or territories and leads to over 4 million deaths a year.

Diabetes mellitus is a progressive and complex metabolic disorder, characterized by chronic hyperglycemia, caused by impaired insulin secretion and (or) utilization. It is currently generally classified into four categories, that is type 1 diabetes mellitus (T1DM), type 2 diabetes mellitus (T2DM), specific types of diabetes mellitus and gestational diabetes mellitus [66,67]. T1DM is usually caused by the autoimmune destruction of β cells, resulting in an absolute deficiency of insulin. T2DM, accounting for 90% of all DM, usually occurs in a context of insulin resistance, with a progressive loss of insulin secretion in β cells [66].

**Figure 2:**
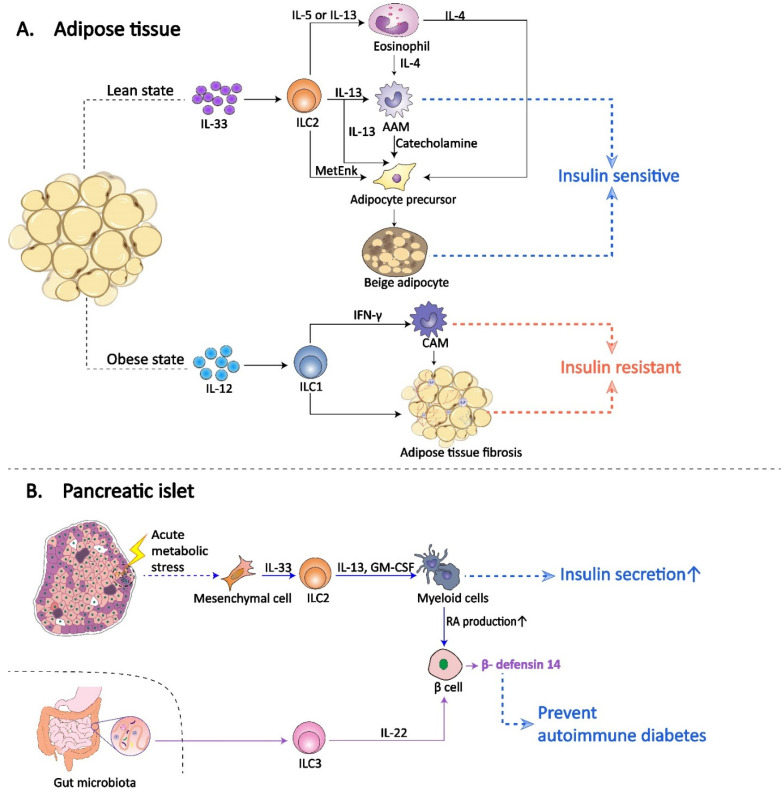
Role of ILCs in diabetes mellitus. A | ILCs in adipose tissue. In the lean state, IL-33 induces adipose-resident ILC2s to produce the cytokines IL-5 or IL-13, which support the recruitment and accumulation of eosinophils in AT. Eosinophils produce IL-4 to sustain and recruit AAMs. ILC2s produce ample IL 13 and may also directly contribute to AAM recruitment and maintenance. AAM byproducts, such as IL-10, contribute to adipocyte insulin sensitivity and protect against DM. In addition, IL-4, IL-13 and methionine-enkephalin peptides (MetEnk) and catecholamines, produced by eosinophils, ILC2s and AAMs, respectively, promote the proliferation and differentiation of adipocyte precursors into beige adipocytes. Beige fat biogenesis also promotes insulin sensitivity and prevents DM. In the obese state, while IL-12 promotes the selective accumulation of adipose-resident ILC1s. ILC1s drive CAM polarization by IFN-γ production and promote AT fibrosis, contributing to obesity-associated insulin resistance and DM. B | ILCs in pancreatic islets. In diabetic or obese states, the islets are also in an inflammatory background. IL-33 is produced by mesenchymal cells as a stress signal in islets. As the main IL-33-responsive cells in islets, islet-resident ILC2s stimulate the capacity of myeloid cells to produce RA, which in turn enhances insulin secretion in islet β cells and protects against DM. In the gut, the microbiota controls IL-22 expression by ILC3s within pancreatic islets through different pathways. ILC3-derived IL-22 induces islet β cells to produce β-defensin, preventing autoimmune diabetes.

ILCs resident in AT have been proven to limit or promote the development of obesity and obesity-associated T2DM (Figure 2). In healthy lean individuals, AT is enriched with type 2 immune cells, such as ILC2s, eosinophils and alternatively activated macrophages (AAMs, anti-inflammatory or M2 macrophages), to maintain tissue homeostasis and support a metabolically healthy state. During the process of type 2 immunity in AT, ILC2 plays an integral role in communication and regulation, and are therefore indispensable regulators. On the one hand, ILC2s produce cytokines IL-5 and IL-13, promoting the recruitment and accumulation of eosinophils and AAMs to support AT remodeling and to restrict “type 1” inflammatory responses [68,69,70,71,72]. On the other hand, ILC2s promote the beiging of white adipose tissue (WAT), contributing to an increasing of both the quantity and the performance of beige adipocytes in AT [73,74,75]. Through the two known mechanisms above, ILC2s help with the maintenance of AT balance and protect from obesity-associated metabolic dysfunction, insulin resistance and T2DM. In the AT of obese patients, diet-induced obesity initiates the early production of IL-12, which results in selective proliferation and accumulation of ILC1s which requires the IL-12 receptor and STAT4 signaling [76]. ILC1-derived IFN-γ is necessary to accelerate classically activated macrophages (CAMs, proinflammatory macrophages or M1 macrophages) polarization and contributes to obesity-associated insulin resistance. In addition, adipose ILC1s have also been demonstrated to promote AT fibrogenesis by increasing M1 macrophages and activating the TGF-β1/Smad3 signaling pathway [77,78]. By recruiting and activating M1 macrophages and inducing AT fibrosis, adipose-resident ILC1s participate in and promote the progression of insulin resistance and obesity-associated diabetes. It is also worth mentioning that AT is an important source of reactive oxygen species (ROS). In the obese state, the AT expands and local chronic inflammation occurs, and ROS production level is also significantly elevated [79]. Poorly protected mitochondrial DNA is sensitive to the oxidative damage and injury of other harmful mutagens. The ROS-mediated oxidative stress can significantly impair the mitochondrial DNA, leading to mitochondrial DNA mutations and epigenetic alterations, thus inducing cancer initiation and progression [80]. As a crucial innate immune cell involved in chronic inflammation of AT under obesity, whether ILCs are involved in ROS production and the subsequent cancer induction may become another issue.

A variety of substances that constitute the diabetic milieu, such as glucose and saturated fatty acids, stimulate the islet to produce various proinflammatory chemokines and cytokines and recruit and activate type 1 immune cells [81,82,83,84]. Therefore, anti-inflammatory drugs for T2DM treatment are under development [85]. Using immunofluorescence analyses, Dalmas et al. confirmed the existence of ILC2s located inside or in the periphery of islets in the mouse pancreas [86]. Under conditions of islet inflammation in T2DM, proinflammatory factors induce mesenchymal cell-derived IL-33. Islet-resident ILC2s expressing the IL-33 receptor (IL-33R) are the major IL-33-responsive cells in islets. ILC2s increase the number of islet myeloid cells and elicit their capacity to promote retinoic acid (RA) production in a manner dependent on the secretion of IL-13 and GM-CSF [87,88]. Ultimately, increased RA in turn enhances insulin secretion in islet β cells. The process above may be associated in part with the phenotypic plasticity shown by ILC2s in response to inflammatory signaling [89,90].

Islet-resident ILC3s have been found to play a role in protecting against autoimmune diabetes in mouse models (Figure 2). This function is mainly achieved by ILC3-induced mouse β-defensin 14 (mBD14) expression, and the activation of the former depends on gut microbiota [91]. Defensin is a kind of antimicrobial peptide whose abnormal expression has been proven to be associated with diseases, including autoimmune diabetes [92,93]. In the gut, the microbiota is known to control IL-22 expression in ILC3s through different pathways. On the one hand, by expressing aryl hydrocarbon receptor (AHR) ligands, some specific gut microbes can directly stimulate ILC3s to produce IL-22 [94,95,96,97]. On the other hand, other gut microbiota indirectly positively affects ILC3s via the induction of IL-23 secretion (a strong inducer of IL-22) by intestinal phagocytes [95]. These pathways also work in islets to stimulate islet-resident ILC3s to secrete IL-22 [91]. In pancreatic islet, ILC3s are the major source of IL-22. ILC3-derived IL-22 induces islet β cells to produce mBD14, preventing autoimmune diabetes through the ILC3-IL22-mBD14 axis. In addition, ILC3s have also been found to produce GM-CSF and thus may play a partial role in regulating insulin secretion and protecting against T2DM [86,87].

## 4. ILCs (Mainly NK Cells) in Pancreatitis

Pancreatitis is an inflammatory disease of pancreatic tissue. Different etiologies, including pancreatic duct obstruction secondary to gallstones, alcohol abuse, as well as surgical trauma or pharmacological means, cause the dysfunction of cellular pathways and organelles, ultimately leading to acinar cell death and local and systemic inflammation [98]. Pancreatitis is one of the most common causes of hospitalization among all gastrointestinal diseases, with a high morbidity, mortality and socioeconomic burden [99,100].

Despite its complex underlying pathophysiology, the pathogenesis and progression of pancreatitis are recognized to have a great deal to do with immune cells [101]. As a recently discovered immune cell group, the role of ILCs in pancreatitis has not been well studied. Current studies are mainly aimed at NK cells, but the knowledge gained is still relatively limited. There are practical challenges in acquiring human pancreatic tissue during the episodes acute pancreatitis (AP), so researchers often use animal models or human peripheral blood to simulate or infer this process. Several clinical studies have shown that the frequency of peripheral NK cells is significantly reduced in the first few days in patients with AP [102,103,104,105]. This may be due to the migration of peripheral NK cells to the inflammatory sites [106]. Dabrowski’s study suggests that depletion of peripheral NK cells in severe acute pancreatitis (SAP) represents the suppressive state of innate immunity, which may be responsible for the secondary infection of AP [103]. Some studies have shown that although the number of peripheral NK cells decreases at the initial stage of SAP, it will subsequently rise back and exceed normal levels, and the over-activation and high-response of NK cells are considered a pathophysiological mechanism for AP progression from local inflammation to systemic inflammatory response syndrome or secondary infection with pancreatic necrosis [105]. There is still no established conclusion whether NK cells play a promoting, inhibitory or irrelevant role in the progression of AP. The lack of human tissue specimens of AP is one of the greatest difficulties. Besides, dynamic monitoring of the number and activity of NK cells, as well as its cytokines, such as TNF-and IL-6, is also necessary. In patients with chronic pancreatitis (CP), a decrease in NK cell number and activity in the peripheral blood has also been observed, especially in those with abdominal pain [102,107]. However, considering that NK cells are not resident in a single lymphoid or nonlymphoid tissue, studies in which the immune function of NK cells in patients with pancreatitis is evaluated through the peripheral blood compartment may still be open to questioning. It is also regrettable that there are no conclusive findings about the certain role of NK cells in the pathophysiology of pancreatitis. For CP, it is also worth focusing our attention to the DM that it causes, which is also called pancreatogenic diabetes. A variety of different pancreatic exocrine diseases may lead to different mechanisms of hyperglycemia, and CP is the most common cause of pancreatogenic diabetes. The prevalence of diabetes secondary to CP is ranges from approximately 25% to 80% [108], and understanding the intrinsic link between CP and the onset of DM is necessary to establish accurate diagnostic criteria and develop effective treatments.

Abnormal expression of CD4^+^ T cells is hypothesized to be responsible for the progression of AP and CP, and is also a critical and sufficient factor for an autoimmune pancreatitis pathogenesis. A Th1 cytokine profile is strongly associated with SAP, while a Th2 profile with mild AP or moderately SAP. Moreover, immune profiles of peripancreatic tissue reveals a Th2 cell-driven anti-inflammatory response [109,110]. Therefore, appropriately reducing the Th1 cell frequency and maintaining the balance of Th1/Th2 ratio may be one of the treatments to prevent the deterioration of AP. During the pathogenesis of AP, IL-17, mainly produced by Th17, is able to recruit neutrophils and macrophages to the inflammatory sites by regulating inflammatory molecules, ultimately leading to a cascade of amplification of inflammatory responses and pancreatic injury [111]. IL-17-induced neutrophils chemoattraction cause pancreatic duct obstruction and subsequent focal inflammation, which also determines the severity of CP [112]. The function of T lymphocytes at the inflammation site and in the peripheral blood has been extensively studied in the development of pancreatitis [113]. As the innate counterparts of helper T cells, whether ILC play a promoting or inhibitory role in the progression of this disease is worth exploration. Therefore, although the knowledge of ILC function in pancreatitis remains unexplored, it is still reasonable to believe studies in this area are meaningful.

## 5. ILCs in Pancreatic Cancer

Pancreatic cancer (PC) is one of the most fatal malignant tumor whose incidence is only 14th among all cancers, while it remains the 7th most common cause of cancer death worldwide [4]. It has become the third leading cause of cancer death in the United States, with the lowest 5-year survival rate among all cancers of 9% [114]. Its characteristics of rapid progression, early metastasis and late diagnosis make PC the recognized “king of cancer”.

**Figure 3:**
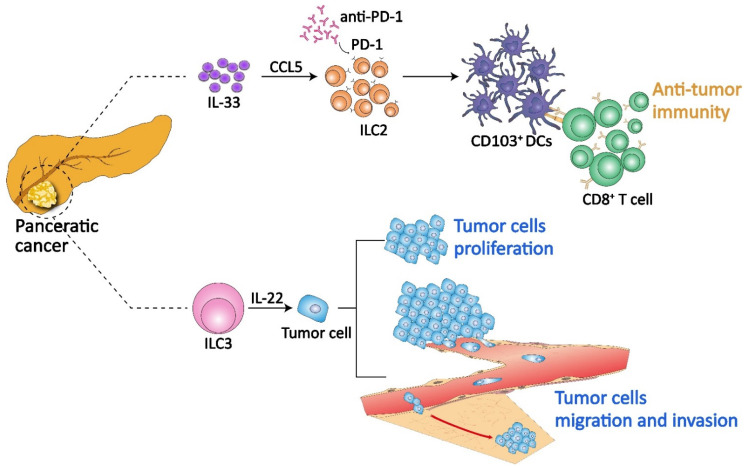
Role of ILCs in pancreatic cancer. ILCs may act as a double-edged sword in pancreatic cancer. In PC tissues, the frequencies of ILC2s and ILC3s are both significantly increased. Expanded by IL-33, ILC2s in PC potentially produce chemokine CCL5, which promote the recruitment and accumulation of CD103^+^ DCs in tumor tissues and further activate antitumor immunity in CD8^+^T cells. ILC2s express the PD-1, which restrains antitumor immunity. However, the PD-1 inhibition on ILC2 can be relieved by antibody-mediated PD-1 blockade, identifying ILC2s to be a potential, promising and brand-new target for anti-PD-1 immunotherapy. Unlike ILC2s, ILC3s promote the proliferation, metastasis and invasion of PC cells through IL-22/AKT signaling.

As crucial participants in innate immunity, ILCs undoubtedly play a role in the early stages of oncogenesis, in the formation of the tumor microenvironment, and in the whole process of tumor progression and metastasis. They sense malignant transformation and promote or inhibit tumor progression by producing an array of cytokines. To date, all known ILC subsets have been identified in PC but are found in different abundances. NK cells are the predominant ILC subset found in PC. They can prevent the growth of pancreatic tumors and induce remodeling of the tumor microenvironment, thus they have recently been targeted for tumor immunotherapy [64,115,116]. In contrast, due the relatively late discovery of them, the inadequate studies on them and the lack of specific markers to identify them, little is known about noncytotoxic “helper” ILCs in pancreatic tumors. In fact, they may play a double-edged sword role in this disease (Figure 3).

The presence of ILCs and ILC2s is detected in specimens from peripheral blood, pancreatic tissue and pancreatic tumor tissues of PC patients using flow cytometry, and higher ILC2 frequency is associated with longer survival [117]. ILC2s may exert an antitumor efficiency in human PC. In KPC mice and orthotopic PC mice established with KPC cell lines, mouse ILC2s with a similar phenotype to human PC ILC2s are also identified, and display a characteristic tissue residency. Studies on the orthotopic mouse models of PC show that ILC2s in the PC microenvironment can activate antitumor immunity and act as targets of anti-PD-1 immunotherapy. Programmed death-1 (PD-1) is a T-cell coinhibitory receptor whose overexpression on tumor cells and tumor-infiltrating lymphocytes correlates with a poor disease outcome and tumor recurrence in many human cancers [118,119,120,121]. Inhibition of the combination and interactions between PD-1 and its ligand PD-L1 can potentiate antitumor activity by enhancing T cell functions, which has been developed for cancer immunotherapy. Recently, PD-1 has also been found to be expressed in ILC2s and plays a negative regulation role in controlling cell proliferation and cytokine expression [122,123]. Expanded by IL-33, ILC2s in PC potentially produce chemokine CCL5, which promote the recruitment and accumulation of CD103^+^ DCs in tumor tissues and further activate antitumor immunity in CD8^+^T cells [117,124]. Similar antitumor activity has also been described in lung ILC2s, as IL-33-activated ILC2s produce cytokines IL-5 and IL-13, recruiting eosinophils and DCs to the tumor site, respectively, which limits tumor growth and invasion [125,126]. However, PD-1 restrains the cell-intrinsic ILC2 functions described above. Further research suggests that antibody-mediated PD-1 blockade can release this PD-1 inhibition in tumor ILC2s, rather than in T cells, to activate antitumor immunity. That is, ILC2s act as tissue-specific enhancers to boost tumor immunity and amplify the therapeutic efficacy of anti-PD-1 in PC [117]. In addition, IL-33 treatment is indicated to upregulate PD-1 expression in a fraction of tumor ILC2s; thus, a combination treatment of recombinant IL-33 and anti-PD-1 may maximally activate and enrich ILC2s in PC and enhance tumor control.

X. Xuan et al. analyzed the data of ILC frequency in pancreas and peripheral blood of PC patients and normal controls by flow cytometry, and they also observed the significantly increased levels of ILC2s and ILC3s in cancer tissues [127]. This phenomenon is consistent with the findings of Moral et al. [117]. By connecting the clinicopathological features of PC patients, it can found that the higher ILC3 frequency in tumor tissue is closely associated with tumor cell proliferation, vascular invasion and distant metastasis in human PC. IL-22, one of the major cytokines by which ILC3s perform their biological and pathological functions, has been found to be associated with the pathogenesis of many cancers, such as lung cancer, hepatocellular carcinoma, gastric cancer and colorectal cancer [128,129,130,131]. A significantly elevated secretion level of IL-22 is also found in PC tissues, and ILC3s are its important source. ILC3s in the PC microenvironment enhance the potential of PC cells for proliferation, invasion and migration, by the combination of IL-22 with its cognate receptor IL-22R and the subsequent activation of the AKT signaling pathway, as is demonstrated by the in vitro experiments by X. Xuan et al. [127].

Although some studies on the role of ILCs in PC have been undertaken, our understanding of this area is still vague. More data remain to be collected, and several important issues need to be addressed. First, PC mouse models constructed by injecting tumor cell lines or some chemical carcinogens have difficulty reproducing a physiological tumor microenvironment. Therefore, biopsy samples of human PC tissues are needed for detailed analysis of these ILC subsets. Furthermore, it is also necessary to develop better molecular tools or detection devices to quantitatively and qualitatively evaluate individual subsets of ILC. In addition, we need to explore the latent power of these cells not merely from their direct impact on PC cells but also from their ability to communicate with different components within the tumor microenvironment [132], which may provide multiple insights into how to effectively manipulate and utilize ILCs for PC therapies.

## 6. Diagnostic and Therapeutic Implications

The identification of ILCs associated with human pancreatic diseases, such as DM, pancreatitis and PC, is still in its infancy. It is hard to say whether the regulation of ILCs and the induction or elimination of their cytokine products could be developed and utilized as part of future treatment. However, some experimental evidence available in mouse studies (Table 1) and human clinical trials (Table 2) may give us some guidance. In Table 1, we list the possible role of ILCs in pancreatic disease demonstrated by animal studies, and propose point-to-point conjectures based on these evidences to discuss the potential value of ILCs in the diagnosis and treatment of pancreatic disease. In Table 2, we present the evidence associated with human clinical trials to discuss the functional and applicable prospects of ILCs.

As mentioned before, adipose-resident ILC2s promote “type 2” inflammatory responses and beiging of WAT [69,70,71,73,74,75], which contributes to the maintenance of the AT balance and protects against obesity-associated diabetes. In contrast, ILC1s strengthen “type 1” inflammatory responses and fibrogenesis in AT, leading to insulin resistance and the development of DM [76,77,135]. Therefore, manipulating ILC subsets in AT, which includes promoting appropriate activation and expansion of ILC2s and inhibiting excessive proliferation of ILC1s may offer new therapeutic avenues for obesity-related diabetes.

Death receptor 3 (DR3) is a member of the tumor necrosis factor receptor superfamily, and has been found to be expressed on the surface of ILC2s. As a specific modulator of ILC2 effector functions, DR3 is able to induce both naïve or activated ILC2 to express type 2 cytokines, thereby protecting against the onset of T2DM [136]. Thus, DR3 agonists may be a novel therapeutic drug for the prevention and treatment of T2DM.

In addition, circulating ILC1 levels are positively associated with some DM clinical parameters, including glycated hemoglobin (HbA1c), serum free fatty acids (FFAs), and so on. And higher ILC1 levels indicate a 13.481-fold greater risk of T2DM [135]. Therefore, circulating ILC1 levels may be used as a good indicator of T2DM. ILC2s resident in pancreatic islet can be activated by IL-33 and promote insulin secretion and prevent T2DM by eliciting the ability of myeloid cells to produce RA. In genetic or diet-induced obese mice, a period of IL-33 treatment controlled and improved glucose homeostasis, suggesting its potential role in T2DM therapy [86]. Moreover, whether the promotion of the ILC3-IL22-mBD14 functional axis in islets by the induction of IL-23 secretion or supplementation with an AHR agonist will help to prevent autoimmune diabetes in humans remains a topic worthy of further discussion [91]. Butyrate, a metabolite derived from gut microbiota, which can upregulate the IL-23 production in DCs and macrophages, has recently achieved good results in mice to protect against autoimmune diabetes [137,138,139,140,141]. Whether the therapeutic effect of butyrate treatment is partly realized by islet-resident ILC3s also deserves future research and discussion.

As an approach to β-cell replacement therapy, pancreatic islet transplantation has shown promise as a therapy for insulin-deficient diabetes and it contributes to the reconstruction of glucose homeostasis. However, islet graft rejection remains a main obstacle to successful transplantation. Due to its severe side effects, there are still limitations to the application of immunosuppressive therapies for the long-term tolerance of islet grafts [142]. In mouse models of islet transplantation, short-term IL-33 treatment has been found to inhibit allogeneic immune responses by the augmentation of Tregs and ILC2s in vivo to prolong islet allograft survival. ILC2s play a particularly important role in this process in an IL-10-dependent manner, and the cotransplantation of ILC2^10^ cells (IL-10-producing ILC2s) with islets can lead to long-term survival after islet transplantation [134]. In the near future, the administration of ILC2^10^ cells as an adjunctive therapy for the prevention of allograft rejection may bring novel therapeutics to islet transplantation. Local delivery of ILC2^10^ cells could become a prospective tool to promote the long-term survival of islet grafts.

As a group of innate immune cells that have only recently entered the field of vision of researchers, concrete studies about the function of “helper” ILCs in pancreatitis have not been formally reported. However, as an early source of cytokines responding to various stimuli and the innate counterparts of helper T cells, whether they play a promoting or inhibitory role in the disease development process is worthy of our future exploration.

ILCs may act as a double-edged sword in the oncogenesis and lesion progression of PC. On the one hand, ILC3s are thought to promote the proliferation, invasion and migration of PC cells through the IL-22/IL-22R-AKT signaling pathways, indicating a latent, promising and brand-new intervention target for PC treatment [127]. Using a neutralizing anti-IL-22 antibody, an anti-IL-22R antibody or an AKT inhibitor to block this signaling pathway may be an effective therapy. On the other hand, ILC2s have emerged as antitumor immune cells for PC treatment and they partly enhance the therapeutic effect of anti-PD-1 immunotherapy [117]. Interrupting the PD-1 signaling pathway that activates tumor-infiltrating ILC2s can promote antitumor effects, and a combination of IL-33 treatment and anti-PD-1 therapy may maximally expand ILC2s and promote their function. In addition, since ILC2s and T cells coexist in human PC and share several immunomodulatory molecules, broader checkpoints can therefore be cotargeted on both ILC2s and T cells in PC. Studies that jointly target ILC2s and T cells in PC immunotherapy are thus warranted.

## 7. Conclusions

Since their discovery and nomenclature consolidation, ILCs have been widely studied. As an important effector cell of innate immunity, ILCs are key players in withstanding pathogen infection, maintaining metabolic homeostasis and participating in tissue remodeling and repair. Although ILCs primarily function in barrier tissues, recent evidence has also indicated that their roles in pancreatic health and disease are by no means negligible. ILCs resident in adipose and pancreatic islets have been proven to be associated with DM, and circulating ILC levels could be used as a good indicator of disease. Furthermore, ILCs may also be involved in the development of pancreatitis and PC. A number of preclinical studies have demonstrated the potential roles of ILCs in the pancreas. However, knowledge in this area is still extremely limited. To date, the developmental lineages and specific characteristics of ILCs in the pancreas have not been fully studied. Their specific localization in the pancreatic tissue, their potential interaction with surrounding immune and parenchymal cells, and their dynamic changes in cell numbers and functions remain unclear. Therefore, further work is necessary to clarify the above queries. Taken together, profound knowledge of ILCs in the pancreas and other related tissues and their complex interactions with other immune or nonimmune cells may provide new treatments for pancreatic diseases.

## Figures and Tables

**Table 1:** Animal studies identifying ILC subsets associated with pancreatic disease and therapy.

Pancreatic Disease or Therapy	ILC Subsets	Located Tissue	The Role of ILCs	Future Perspective for Application and Treatment	Reference
Diabetes mellitus	NK cell, ILC1	Adipose tissue	Driven by IL-12 and STAT4 signaling, adipose NK cells and ILC1s proliferate and accumulate, contributing to obesity-related insulin resistance.	Blocking the IL-12/IL-12R/STAT4 signaling pathway in adipose NK cells and ILC1s may prevent the occurrence of type 2 diabetes mellitus.	[76]
Diabetes mellitus	NK cell, ILC1	Adipose tissue	Lnk/Sh2b3 gene regulate the IL-15/JAK3/STAT5 signaling pathway in adipose NK cells and ILC1s to inhibit the cell number and activity, thereby reducing the risk of diabetes mellitus. Missense variants of Lnk/Sh2b3 gene may contribute to diabetes mellitus.	Blocking the IL-15/JAK3/STAT5 signaling pathway in adipose NK cells and ILC1s may prevent diabetes mellitus caused by Lnk/Sh2b3 gene missense variation.	[133]
Diabetes mellitus	ILC2	Adipose tissue	IL-33 is required to maintain the ILC2s in the white adipose tissue, and ILC2s promote the beiging of white adipose tissue and limit obesity and obesity-related diabetes mellitus.	Providing or maintaining adequate IL-33 and ILC2s to promote beiging of white adipose tissue may be a novel approach to prevent or treat obesity-associated diabetes mellitus.	[73]
Diabetes mellitus	ILC2	Pancreatic islet	IL-33-activated islet-resident ILC2s promote insulin secretion. However, IL-33-ILC2 axis is defective in islets during obesity and is activated following acute β cell stress.	Selectively activation of IL-33-ILC2 axis in islet may offer therapy for diabetes mellitus.	[86]
Diabetes mellitus	ILC3	Pancreatic islet	Gut microbiota-regulated islet-resident ILC3s secrete IL-22 to support pancreatic endocrine cells to express β-defensin 14, preventing autoimmune diabetes.	Increasing the secretion of ILC3-derived IL-22 in islets via the intestinal pathways may prevent autoimmune diabetes.	[91]
Pancreatic cancer	ILC2	Pancreas	ILC2s emerge as antitumor immune cells for pancreatic cancer treatment and partly enhance the therapeutic effect of anti-PD-1 immunotherapy.	Blocking PD-1 signaling pathway on tumor-infiltrating ILC2s may promote antitumor effects and pancreatic cancer immunotherapy.	[117]
Islet transplantation	ILC2	Pancreatic islet	ILC2s prolong islet allograft survival in an IL-10-dependent manner.	Local delivery of ILC2^10^ could be a promising tool to promote long-term islet graft survival.	[134]

**Table 2:** Human clinical studies identifying ILC subsets associated with pancreatic disease and their possible roles.

Pancreatic Disease	ILC Subsets	Located Tissue	The Role of ILCs	Future Perspective for Application and Treatment	Reference
Diabetes mellitus	ILC1	Adipose tissue	ILC1s promote adipose tissue fibrosis and diabetes mellitus in obesity.	Inhibiting the accumulation of adipose ILC1s may attenuate adipose tissue fibrogenesis and protect against type 2 diabetes mellitus.	[77]
Diabetes mellitus	ILC1	Peripheral blood	In patients with type 2 diabetes mellitus, ILC1s are significantly increased in the peripheral blood, and a higher ILC1 level indicates a 13.481-fold greater risk of diabetes mellitus.	Circulating ILC1s can be a good indicator of type 2 diabetes mellitus.	[135]
Diabetes mellitus	ILC2	Peripheral blood	DR3 induce human ILC2s to express type 2 cytokines and prevent type 2 diabetes mellitus.	DR3 agonist may be a novel, promising and worth exploring therapeutic avenue for type 2 diabetes mellitus.	[136]
Pancreatitis	NK cell	peripheral blood	In acute pancreatitis patients, NK cell frequency correlates positively with amylase and lipase concentration, as well as the length of hospital stay.	Changes of NK cell level in peripheral blood can act as an auxiliary diagnosis indicator for acute pancreatitis.	[102]
Pancreatic cancer	ILC2	pancreas	PD-1^+^ tumor ILC2s and PD-1^+^ T cells coexist in nearly 60% of human pancreatic ductal adenocarcinomas and show a significant correlation.	ILC2s may be conducive to the clinical curative effect of PD-1 therapy.	[117]
Pancreatic cancer	ILC3	pancreas	Higher ILC3 frequency in tumor tissue is closely associated with tumor cell proliferation, vascular invasion, and distant metastasis in human pancreatic cancer. Subsequent in vitro experiments demonstrated that ILC3s promote the pancreatic cancer development through IL-22/IL-22R-AKT signaling pathway.	Use of neutralizing IL-22 antibody, IL-22R antibody or AKT inhibitor to block the IL-22/IL-22R-AKT signaling pathway may be an effective therapy for pancreatic cancer.	[127]

## Data Availability

Not applicable.
